# Predictors and disturbances of sleep quality between men and women: results from a cross-sectional study in Jordan

**DOI:** 10.1186/s12888-024-05662-x

**Published:** 2024-03-12

**Authors:** Mohammad R. Alosta, Islam Oweidat, Mohammad Alsadi, Mahmoud Mohammad Alsaraireh, Bayan Oleimat, Elham H. Othman

**Affiliations:** 1 Faculty of Nursing, Zarqa University, Zarqa, Jordan; 2 Nursing Administration & Education Department, Faculty of Nursing, Jouf University, Sakaka, Saudi Arabia; 3 Prince Aisha Bint Alhussein College for nursing and health sciences, Alhussein Bin Talal University, Ma’an, Jordan; 4 Faculty of Nursing, Applied Science Private University, Amman, Jordan

**Keywords:** Gender, Men, Pittsburgh Sleep Quality Index, Sleep, Sleep quality, Survey, Women

## Abstract

**Background:**

Sleep disturbances, a public health concern that may lead to critical physiological conditions, are associated with personal characteristics such as gender. Limited evidence is available from the Middle East population on the gender disparities in sleep quality. Therefore, the current study examined gender-specific differences in sleep quality and disturbances among Jordanian citizens.

**Method:**

A cross-sectional design was used to recruit a convenient sample of 1,092 adults from different Jordanian cities. Data was collected using a self-reported questionnaire comprising the Pittsburgh Sleep Quality Index (PSQI), which was distributed online via social media networks. The participants were categorized according to their global PSQI scores into poor (PSQI ≥ 5) and good sleepers (PSQI < 5). The analysis focused on finding differences between women and men in terms of sleep quality and the effects of demographic, lifestyle, and socioeconomic factors on reported sleep problems.

**Results:**

Women were revealed to have a higher prevalence of all types of sleep disturbances than men. Women who were over 55 (compared to younger than 20 years), did not smoke, had multiple jobs or part-time employment (compared to unemployed women), and had a monthly income of more than 500 JD (compared to those with an income of < 500 JD) were less likely to experience poor sleep than other women. In contrast, men who neither smoked nor drank coffee, ate no sweets or only one to two pieces daily (compared to participants who ate more than two pieces daily), and worked fixed night shifts (compared to alternating shifts workers) were less likely to experience poor sleep than other men.

**Conclusion:**

This study builds a more nuanced understanding of how different demographic, lifestyle, and socioeconomic factors - such as a participant’s age, time of working duty, income, daily sweet consumption, daily caffeine consumption, and smoking - affect the sleep quality of men and women. Thus, promoting a healthier lifestyle for both genders by modifying risk factors - such as smoking cessation, as well as reducing their intake of caffeine and sweets - is the first step toward improving their sleep quality. Further studies are needed to examine how the social role of Arabic women affects their sleep.

## Background

Nowadays, having insufficient good-quality sleep has become a public health concern, and it is considered one of the main factors affecting the general health status and well-being of individuals and communities [1]. The literature indicates that sleep disturbances, including insomnia, may lead to various critical physiological conditions like myocardial infarction and stroke [2, 3], in addition to mental disorders like anxiety and depression [1]. Globally, several factors can impede or enhance sleep quality, including age, gender, and levels of physical activity. More specifically, sleep disturbances are associated with personal characteristics such as gender [4–6].

In terms of gender-based differences in sleep quality, previous studies have reported the impacts of several factors on sleep quality for men and women at various life stages. For instance, a study reported that a high sweets consumption affected women’s sleep quality more than that of men [7]. Moreover, other studies showed that caffeine intake is more frequent in females predisposing them to sleep disturbances and short sleep duration [8]. Furthermore, despite the low tobacco and alcohol consumption among women, they still suffered more frequent sleep problems than men [9].

Another study on young adults demonstrated that physical exercise improved sleep quality among male university students, while it did not affect the sleep quality of their female equivalents [4]. Another study reported that women over 30 years of age tend to have lower sleep quality, which thereafter deteriorates faster compared to the rate among men of a similar age [5]. A similar study revealed that sleep latency only increases with age in women, while sleep efficiency reduces in both genders as they age [10].

Arab sociocultural factors can influence various aspects of life, including sleep patterns and habits. For instance, it is not uncommon for extended families to live together, which may affect sleep patterns because individuals may be influenced by the schedules and activities of other family members. Moreover, Arab cultures often place a high value on socializing and hospitality. Late-night gatherings, especially during weekends and special occasions, may lead to later bedtimes [11]. Further, sleep problems are influenced by the different social roles of men and women. For instance, women’s sleep is affected by their job requirements and family responsibilities, while men’s sleep is more likely to be disrupted only by their job requirements [12, 13]. Furthermore, due to cultural and social norms, Arabic women typically have additional duties alongside their employment, which may increase their stress levels and consequently affect their sleep quality [14–16].

It can be inferred that the varied and complicated reports in the literature support the development of specific interventions for different groups of the population, among which gender-specific interventions are recommended [2, 4]. Limited evidence is available from the Middle East population on gender disparities in sleep quality. Therefore, the current study aimed to examine gender-specific differences in sleep quality and sleep disturbances among Jordanians, as well as identify predictors of their sleep quality.

## Methods

### Design and data collection

Based on a cross-sectional design, convenience sampling was utilized to recruit Jordanian citizens from different governorates across the northern, central, and southern regions of the country. All Jordanian citizens above 18 years old who could read and comprehend Arabic were eligible to participate in the study without other restrictions.

The current study is a part of a major study examining different lifestyle behaviors among Jordanians. Data was collected using a self-reported Google Form questionnaire distributed online via social media networks such as Facebook (on public groups and pages) and WhatsApp (through the researchers’ social networks) between April and September 2022. The initial questionnaire was composed by the researchers, items were selected after reviewing similar literature, and the survey included the International Physical Activity Questionnaire - Short Form and the Pittsburgh Sleep Quality Index (PSQI). Answering all the questions was mandatory, but the participants could review questions and modify their answers as necessary.

The questionnaire for the current paper comprised a demographic data sheet including personal characteristics, daily consumption of sweets and caffeinated drinks, and smoking status (the participant was considered a smoker if he/she smoked any type of tobacco regularly). The participant’s physical activity during the previous seven days was categorized as health-enhancing physical activity (HEPA) active (at least 1.5-2 h of being active throughout the day), minimally active (at least 20 min of activity on three or more days per week), or inactive.

Sleep quality was measured using the Arabic version of the the PSQI [17]. The PSQI examines sleep quality, sleep latency (time needed to fall asleep), sleep duration (time of actual sleep), habitual sleep efficiency, sleep disturbances (waking up in the middle of the night or early morning, having to get up to use the bathroom, being unable to breathe comfortably, coughing or snoring loudly, feeling too cold, or too hot, having bad dreams, and having pain), use of sleeping medication, and daytime dysfunction. The total scores from the seven PSQI components are calculated to reveal a minimum score of 0 (better sleep) and a maximum score of 3 (worse sleep). Similarly, the global score is calculated by summing the seven components to reveal a score ranging from 0 (better sleep) to 21 (worse sleep), with a cut-off point of 5; a total score of 5 or greater indicates poor sleep quality [18]. The Arabic version of the PSQI has demonstrated adequate reliability; the Cronbach’s α ranged from 0.634 [19] to 0.77 [20]. In the current study, the Cronbach’s α for the PSQI was 0.723.

### Ethical considerations

This research was performed in accordance with the Declaration of Helsinki. Ethical approval was obtained before the study from the Institutional Review Board (IRB) of Zarqa

University, IRB number (20/2021). On the invitation page, participants were informed that they should participate freely without coercion and reminded of their right to withdraw from the study at any time. They were also notified that responding to the electronic questionnaire was regarded as consenting to participate. The anonymous data was downloaded from the Google Form and stored on the researcher’s personal computer with a password lock. No one had access to the data except for the first and corresponding authors. The data was already anonymized, and the respondents were not identified in any way.

### Data analysis

The data was analyzed using IBM SPSS Statistics (version 26). Descriptive statistics were used to describe the study participants and report their sleep quality and disturbances. The differences between men and women in terms of the PSQI components and global scores were examined using T-testing. Chi-square statistics were used to examine which frequencies were statistically significantly different between men and women. The participants were categorized according to their global PSQI scores into poor (PSQI ≥ 5) and good sleepers (PSQI < 5). Then, logistic regression was used to examine the effects of five significant variables -demographic (age), lifestyle (caffeine consumption and smoking), and socioeconomic (employment status and monthly income) - on reported sleep problems among women, as well as the effects of five significant variables: demographic (age), lifestyle (sweet consumption, caffeine consumption, and smoking), and socioeconomic (time of working duty) - on reported sleep problems among men.

## Results

A total of 1,092 participants replied to the electronic link. A full description of the participants is presented in Table (1). Generally, they ranged in age from 20 to 34 (*n* = 555, 50.8%), were women (*n* = 714, 65.4%), and were married (*n* = 696, 63.7%). Regarding employment conditions, most had full-time jobs (*n* = 696, 63.7%), and 55% of the employed participants (*n* = 423) had daytime jobs.

**Table 1:** Demographic characteristics of the participants (*N* = 1092)

	Characteristics	Total *n* (%) *N* = 1092	Women *n* (%) *N* = 714	Men *n* (%) *N* = 378
**Age**	18–19 years	45 (4.1)	33 (4.6)	12 (3.2)
	20–34 years	555 (50.8)	363 (50.8)	192 (50.8)
	35–54 years	474 (43.4)	309 (43.3)	165 (43.7)
	Older than 55	18 (1.6)	9 (1.3)	9 (2.4)
**Marital Status**	Single	360 (33)	240 (33.6)	120 (31.7)
	Married	696 (63.7)	441 (61.8)	255 (67.5)
	Widowed	9 (0.8)	6 (0.8)	3 (0.8)
	Divorced	27 (2.5)	27 (3.8)	0
**Educational Level**	Secondary school	36 (3.3)	30 (4.2)	6 (1.6)
	Diploma	162 (14.8)	105 (14.7)	57 (15.1)
	Bachelor’s degree	693 (63.5)	453 (63.4)	240 (63.5)
	Higher education	201 (18.4)	126 (17.6)	75 (19.8)
**Employment type**	Unemployed	327 (29.9)	243 (34)	84 (22.2)
	Part-time job	33 (3)	18 (2.5)	15 (4)
	Full-time job	696 (63.7)	438 (61.3)	258 (68.3)
	More than one job	36 (3.3)	15 (2.2)	21 (5.6)
Time of duty *	Day time job	423 (55)	312 (66.2)	111 (37.8)
	Nighttime job	42 (5)	27 (5.8)	15 (5.1)
	Day/ Night job	300 (40)	132 (28)	168 (57.1)
**Monthly income**	Less than 500 JD	333 (30.5)	234 (32.8)	99 (26.2)
	500–999 JD	549 (50.3)	351 (49.2)	198 (52.4)
	1000–1500 JD	129 (11.8)	87 (12.2)	42 (11.1)
	More than 1500 JD	81 (7.4)	42 (5.9)	39 (10.3
**Type of community**	Urban	924 (84.6)	636 (89.1)	288 (76.2)
	Rural	168 (15.4)	78 (10.9)	90 (23.8)
**Physical activity**	Inactive	509 (46.6)	371 (51.9)	138 (36.5)
	Minimally active	426 (39)	290 (40.6)	136 (35.9)
	Active	157 (14.4)	53 (7.5)	104 (27.6)
**Smoking**	Not smoker	714 (65.4)	558 (78.2)	156 (41.3)
	Smoker	378 (34.6)	156 (21.8)	222 (58.7)
**Cigarettes consumption** §	Less than 10 / day	219 (57.9)	123 (78.9)	96 (43.3)
	10–20 / day	108 (28.6)	18 (11.5)	90 (40.5)
	More than 20 / day	51 (13.5)	15 (9.6)	36 (16.2)
**Sweet consumption**	No consumption	189 (17.3)	117 (16.4)	72 (19)
	1–2 pieces /day	657 (60.2)	402 (56.3)	255 (67.5)
	More than 2 pieces /day	246 (22.5)	195 (27.3)	51 (13.5)
**Caffeinated drinks consumption**	No consumption	207 (19)	129 (18.1)	78 (20.6)
	1–2 cups /day	600 (54.9)	393 (55)	207 (54.8)
	more than 2 cups /day	285 (26.1)	192 (26.9)	93 (24.6)

* Calculated from the total number of employed participants (*N* = 765); §: Numbers and frequencies are calculated from smokers (*N* = 378)

### Components of the PSQI

The most frequently reported duration needed to fall asleep was within 15 min (*n* = 396, 36.3%), with seven being the most commonly reported sleeping hours (*n* = 426, 39%). The majority affirmed they did not take medication in order to sleep (*n* = 774, 70.9%), and 45% (*n* = 495) of the participants rated their sleep quality as fairly good, in that it did not affect their daytime functioning (*n* = 555, 50.8%), including having trouble staying awake while driving, eating meals, or engaging in social activity. Furthermore, the total scores from the seven PSQI components and the global score were calculated (see Table 2). In general, the global score for women was significantly higher (indicative of poor sleep) than for men, as were all the PSQI components except sleep efficiency.

**Table 2:** Pittsburgh Sleep Quality Index (PSQI): component and global scores (*n* = 1092)

Component			M (SD)			t-test	*p*-value	Median (IQR)		*MW*	*p*-value
			Total sample	Women	Men			Women	Men		
Component-1: Sleep quality			1.30 (1.03)	1.40 (1.02)	1.11 (1.02)	4.41	0.00	1 (1–2)	1(0–2)	7.05	.008
Component-2: Sleep latency			1.49 (0.96)	1.54 (0.97)	1.39 (0.94)	2.43	0.015	2 (1–2)	1 (1–2)	16.53	<.001
Component-3: Sleep duration			1.20 (1.08)	1.15 (1.09)	1.30 (1.06)	-2.17	0.03	1 (0–2)	2 (0–2)	6.10	.014
Component-4: Sleep efficiency			0.20 (0.52)	0.22 (0.56)	0.17 (0.43)	1.68	0.09	0	0	.31	.580
Component-5: Sleep disturbances			1.41 (0.68)	1.51 (0.67)	1.23 (0.65)	6.65	0.00	1 (1–2)	1 (1–2)	41.46	<.001
Component-6: Use of sleeping medication			0.46 (0.81)	0.53 (0.89)	0.32 (0.62)	4.65	0.00	0 (0–1)	0	7.90	.005
Component-7: Daytime dysfunction			0.81 (0.98)	0.88 (1.01)	0.68 (0.91)	3.21	0.00	1 (0–2)	0 (0–1)	9.23	.002
***PSQI global score***:			6.87 (3.80)	7.23 (3.86)	6.20 (3.57)	4.30	0.00	7 (4–9)	6 (4–8)	10.65	.001

### Sleep disturbances by gender

**Fig. 1:**
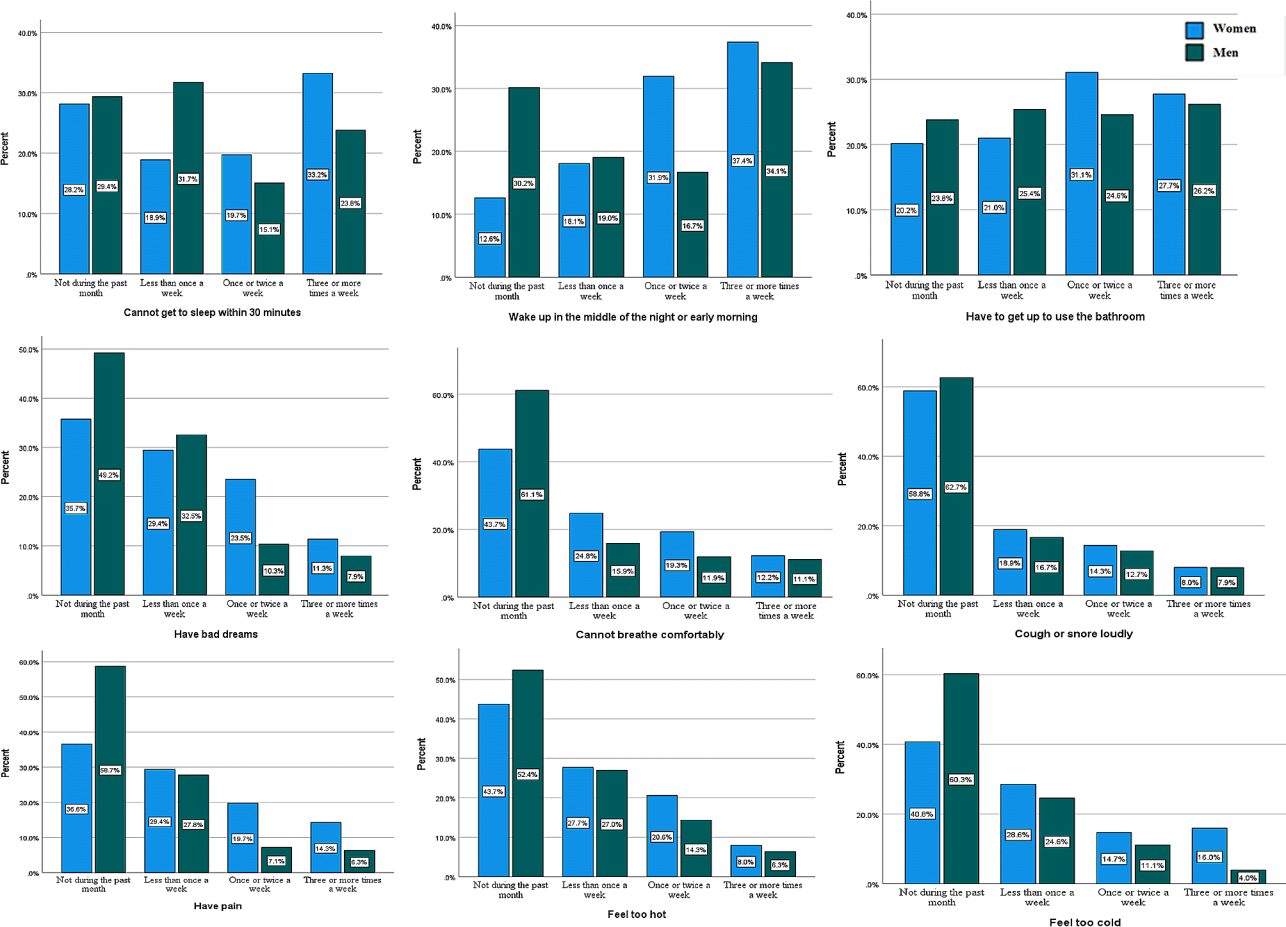
Frequencies of sleep disturbances between men and women

To understand the gender-specific differences in sleep disturbances among the participants, their responses to question 5: “*During the past month, how often have you had trouble sleeping because you*…” were analyzed and categorized based on gender, as displayed in Fig. (1). Overall, women reported a higher prevalence of all types of disturbances than men. More women could not get to sleep within 30 min, *X*^2^ (3, *N* = 1092) = 27.81, *p* < 0.001; woke up in the middle of the night or early morning, *X*^2^ (3, *N* = 1092) = 63.23, *p* < 0.001; could not breathe comfortably, *X*^2^ (3, *N* = 1092) = 32.49, *p* < 0.001; felt too cold, *X*^2^ (3, *N* = 1092) = 53.82, *p* < 0.001; felt too hot, *X*^2^ (3, *N* = 1092) = 10.26, *p* = 0.016; had bad dreams, *X*^2^ (3, *N* = 1092) = 37.51, *p* < 0.001; and had pain *X*^2^ (3, *N* = 1092) = 66.72, *p* < 0.001.

### Risk factors of sleep quality by gender

The logistic regression model for poor sleep among women was statistically significant: *χ*^2^ (13) = 75.05, *p* < 0.001 (Table 3). The model explained 15% (Nagelkerke R^2^) of the variance in sleep quality and correctly classified 74.8% of cases. Women above 55 were less likely to experience poor sleep (OR = 0.107, CI: 0.020–0.566) than participants younger than 20. Similarly, those with incomes of 500–999 JD or 1000–1500 JD had odd ratios of 0.427 (CI: 0.270–0.676) and 0.334 (CI: 0.176–0.632), respectively, for poor sleep, below the value obtained for participants with an income of < 500 JD. Similarly, women who worked part-time (OR = 0.093, CI: 0.028–0.304) and those who had more than one job (OR = 0.139, CI: 0.041–0.476) were less likely to experience poor sleep than unemployed women. Lastly, female smokers were 2.196 times more likely to suffer from poor sleep than female non-smokers (CI: 1.249–3.859).

**Table 3:** Logistic Regression of poor sleep among women

	OR	95% C.I.	*p*-value
**Age**			
< 20 years (Reference)	1		
20–34 years	0.744	0.303–1.828	0.519
35–54 years	0.839	0.323–2.177	0.718
≥ 55 years	0.107	0.020 − 0.566	0.009
**Monthly income**			
Less than 500 JD (Reference)	1		
500–999 JD	0.427	0.270 − 0.676	< 0.001
1000–1500 JD	0.334	0.176 − 0.632	< 0.001
More than 1500 JD	0.693	0.258–1.859	0.466
**Daily consumption of caffeinated drinks**			
None (Reference)	1		
1–2 cups /day	0.722	0.436–1.198	0.208
More than 2 cups /day	1.086	0.597–1.977	0.787
**Employment type**			
Unemployed (Reference)	1		
Part-time job	0.093	0.028 − 0.304	< 0.001
Full-time job	1.128	0.708–1.798	0.611
More than one job	0.139	0.041 − 0.476	0.002
**Smoking status**			
Not smoker (Reference)	1		
Smoker	2.196	1.249–3.859	0.006

JD: Jordanian Dinars

The logistic regression model for having poor sleep among men was statistically significant: *χ*^2^ (12) = 70.13, *p* < 0.001 (Table 4). The model explained 23% (Nagelkerke R^2^) of the variance in sleep quality and correctly classified 69% of cases. Men working fixed night shifts were less likely to experience poor sleep (OR = 0.140, CI: 0.035–0.559) than participants alternating between day and night shifts. Those who ate no sweets or ate one to two pieces per day had odd ratios of 0.236 (CI: 0.084–0.659) and 0.333 (CI: 0.129–0.856), respectively, for having poor sleep, below the values obtained for participants who ate more than two pieces daily. Consuming caffeinated drinks increased the likelihood of poor sleep among men; compared to participants who consumed no caffeine at all, those who drank one to two cups per day were 2.073 times more likely to suffer from poor sleep (CI: 1.132–3.795). Meanwhile, those drinking more than two cups daily were 4.828 times more likely to suffer from poor sleep (CI: 2.210-10.548). Male smokers were 3.110 times more likely to suffer from poor sleep than male non-smokers (CI: 1.657–5.840).

**Table 4:** Logistic Regression of poor sleep among men

	OR	95% C.I.	*p*-value
**Hours of duty**			
Day/Night job (Reference)	1		
Day time job	0.842	0.475–1.491	0.555
Nighttime job	0.140	0.035 − 0.559	0.005
**Daily sweets consumption**			
More than 5 pieces /day (Reference)	1		
1–5 /day	0.333	0.129 − 0.856	0.023
None	0.236	0.084 − 0.659	0.006
**Daily consumption of caffeinated drinks**			
None (Reference)	1		
1–2 cups /day	2.073	1.132–3.795	0.018
More than 2 cups /day	4.828	2.210 -10.548	< 0.001
**Smoking status**			
Not smokers (Reference)	1		
Smokers	3.110	1.657–5.840	< 0.001

## Discussion

This study examined data from 1,092 participants to describe sleep quality among Jordanian citizens and determine the gender-specific differences in their sleep disturbances. The findings revealed that women experienced poor sleep more frequently than men. Given the cultural and social influences involved, traditional gender roles and expectations may affect women’s sleep patterns. Family responsibilities, household duties, as well as attending and serving meals at daily gatherings may impact the time available for women to sleep. Besides, women may experience changes in sleep routines and sleep quality due to caregiving responsibilities [21, 22].

Our findings appear to correspond to previous results. Madrid-Valero and colleagues [23] found that women were almost twice as likely as men to have poor sleep quality. Another study reported that women were 1.4 times more predisposed to insomnia than men [24]. These gender differences might be due to hormonal or physiological changes [25]. Moreover, the current results revealed that women had a higher prevalence of all sleep disturbances than men (being unable to get to sleep within 30 min, waking up in the middle of the night or early morning, being unable to breathe comfortably, feeling too cold or too hot, having bad dreams, and having pain). These results are consistent with those of a previous study by Kerkhof [25], who found that female adolescents reached the highest prevalence rates for most sleep disorders, insufficient sleep, and daytime malfunctioning. Possible explanations could be the physiological and hormonal changes that take place in women’s life phases, as well as the ovarian steroid fluctuations [25, 26].

Examining the influence of demographic, lifestyle, and socioeconomic variables on reported sleep problems in women revealed that those over 55 were less likely to experience poor sleep than their counterparts younger than 20. This aligns with the findings of Miguez and colleagues [27], who found that sleep problems were more prevalent among young adolescents than older women, possibly due to the former’s overuse of technology, video games, and studying with peers. Similarly, participants with an income of more than 500 JD had better sleep than those with lower incomes. This result is congruent with previous results revealing that lower incomes were associated with more sleep complaints [28], which might be related to financial burdens and concerns about life expenses.

Women who worked part-time and those who had more than one job were less likely to experience sleep disturbances than unemployed women. This supported the results obtained by Grandner and colleagues [28], who detected that employment status was associated with fewer sleep complaints. This could be because employed personnel have fixed daily routines, and their working duties consume their energy, leading to their falling asleep without complaints due to exhaustion. Moreover, women smokers were more likely to suffer from poor sleep than female non-smokers; this aligns with another study’s results [29], in which poor sleep quality and sleep disturbances were found to be more common among smokers. This emphasizes how the impact of nicotine consumption on sleep quality unfolds when sleep is attempted.

Meanwhile, men working fixed night shifts were less likely to experience poor sleep compared to those alternating between day and night shifts; this finding is congruent with a previous study in which sleep problems were found to be more frequent among shift workers compared to non-shift workers [30]. This could be because alternating day and night shifts interact with the biological circadian rhythm, resulting in sleep disturbances.

Men who ate fewer sweets reported better sleep than those who ate sweets more frequently; this finding aligns with the results of another study in which poor sleep quality was found to be significantly associated with higher sugar intake [7, 31]. Certain sugary treats such as soda, energy drinks, and juice with sugar will further undermine a person’s sleep, especially if consumed in the evenings [32].

In addition, drinking caffeinated drinks increased the likelihood of poor sleep among men, compared to participants who consumed no caffeine. This result aligns with the study by Sanchez and colleagues [33], who found that men consuming more caffeinated beverages experienced poor sleep quality. A biologically plausible explanation is that consuming caffeinated drinks more frequently reduced homeostatic sleep pressure and decreased the slow wave power in the brain’s frontal, central, and parietal regions, thus affecting the quality and patterns of sleep.

Lastly, the current results reveal that male smokers were more likely to suffer from poor sleep than non-smokers, supporting the results of Liao and colleagues [29]. The latter study found that smokers demonstrated poor sleep quality and sleep disturbances, which were inversely associated with cigarettes smoked per day. This could be due to the physiological desire for additional nicotine during sleep. which may cause smokers to awaken, leading to insomnia. In addition, smoking may affect sleep quality by increasing the risk of snoring and obstructive sleep apnea. Furthermore, considering that nicotine is a stimulant that increases alertness [34], consuming it near bedtime may affect sleep latency.

### Strengths and limitations

The strengths of the present study include the use of valid and reliable tools, as well as sufficient sample sizes and data to discern sleep duration. Although we did not perform sample size calculation for this specific paper- as it was derived from a previous major study, a larger sample size generally results in more precise estimates of population parameters. However, certain limitations affected the work. The online sampling and data collection prevented participation among those without internet access, introducing a selection bias and affecting the generalizability. Additionally, using a self-reported questionnaire may present response biases such as social desirability.

### Implications and recommendations

The current study highlights two serious problems experienced by women that may negatively affect their quality of life: poor sleep quality and a higher prevalence of sleep disturbances than men. Healthcare providers, employers, and family members should consider the sleep quality differences between women and men to provide each gender with appropriate support and help. Identifying sleep disturbances and unhealthy behaviors that negatively affect sleep would improve sleep patterns and assist healthcare providers in promoting healthy lifestyles. These results could guide health promotion programs by focusing on factors that impact sleep quality in women. Future research should emphasize longitudinal analysis to examine how sleep varies over time according to gender. Also required is a qualitative study describing the experience of Arabic women by highlighting the impacts of social and cultural factors on their sleep quality.

## Conclusion

Sleep quality is an important issue throughout life for both men and women. It appears to positively impact health conditions, the ability to function during daytime, and work-related productivity. The results of this study show that the prevalence of poor sleep is considerably higher among women than men; overall, women reported a higher prevalence of all types of sleep disturbance than men. Women were more likely to be unable to sleep within 30 min, wake up in the middle of the night or early morning, be unable to breathe comfortably, feel too cold or too hot, have bad dreams, and have pain. Furthermore, this study builds a more nuanced understanding of how demographics, lifestyle, and socioeconomic factors affect sleep quality among Jordanian citizens, reflecting that gender differences lead to different experiences of and variations in sleep quality. Thus, the first step toward improving sleep quality for both groups is to promote healthy lifestyles among both genders through smoking cessation, as well as reducing the intake of caffeine and sweets. Understanding the sociocultural factors that affect sleep is crucial for healthcare professionals when addressing sleep-related issues in Arabic women. Tailoring interventions to consider cultural nuances and individual experiences can be essential in promoting healthy sleep habits in diverse communities.

## Data Availability

The datasets used and/or analyzed during the current study are available from the corresponding author on reasonable request.

## Declarations

The study was approved by the Research Ethics Committee at the University of Zarqa.

The informed consent was obtained from all the participants.
